# Enhanced lateral flow testing strategies in care homes are associated with poor adherence and were insufficient to prevent COVID-19 outbreaks: results from a mixed methods implementation study

**DOI:** 10.1093/ageing/afab162

**Published:** 2021-07-16

**Authors:** John S P Tulloch, Massimo Micocci, Peter Buckle, Karen Lawrenson, Patrick Kierkegaard, Anna McLister, Adam L Gordon, Marta García-Fiñana, Steve Peddie, Matthew Ashton, Iain Buchan, Paula Parvulescu

**Affiliations:** Institute of Infection, Veterinary and Ecological Sciences, University of Liverpool, Liverpool CH64 7TE, UK; NIHR London In Vitro Diagnostics Co-operative, Department of Surgery and Cancer, Imperial College London, London W2 1NY, UK; NIHR London In Vitro Diagnostics Co-operative, Department of Surgery and Cancer, Imperial College London, London W2 1NY, UK; Public Health Department, Liverpool City Council, Liverpool L3 1DS, UK; NIHR London In Vitro Diagnostics Co-operative, Department of Surgery and Cancer, Imperial College London, London W2 1NY, UK; CRUK Convergence Science Centre, Institute for Cancer Research & Imperial College London, London SW7 2AZ, UK; NIHR London In Vitro Diagnostics Co-operative, Department of Surgery and Cancer, Imperial College London, London W2 1NY, UK; Division of Medical Sciences and Graduate Entry Medicine, University of Nottingham, Nottingham, UK; NIHR Applied Research Collaboration East Midlands (ARC-EM), Nottingham, UK; Institute of Population Health, University of Liverpool, Liverpool, UK; Adults Social Care Department, Liverpool City Council, Liverpool L3 1DS, UK; Public Health Department, Liverpool City Council, Liverpool L3 1DS, UK; Institute of Population Health, University of Liverpool, Liverpool, UK; Public Health Department, Liverpool City Council, Liverpool L3 1DS, UK

**Keywords:** lateral flow devices, antigen test, SARS-CoV-2, care homes, COVID-19, rapid point-of-care testing

## Abstract

**Introduction:**

Care homes have been severely affected by the SARS-CoV-2 pandemic. Rapid antigen testing could identify most SARS-CoV-2 infected staff and visitors before they enter homes. We explored implementation of staff and visitor testing protocols using lateral flow devices (LFDs).

**Methods:**

An evaluation of a SARS-CoV-2 LFD-based testing protocol in 11 care homes in Liverpool, UK, including staff and visitor testing, plus a qualitative exploratory study in nine of these homes. The proportion of pilot homes with outbreaks, and outbreak size, were compared to non-pilot homes in Liverpool. Adherence to testing protocols was evaluated. Fifteen staff were interviewed, and transcript data were thematically coded using an iterative analysis to identify and categorize factors influencing testing implementation.

**Results:**

In total, 1,638 LFD rapid tests were performed on 407 staff. Protocol adherence was poor with 8.6% of staff achieving >75% protocol adherence, and 25.3% achieving $\ge$50%. Six care homes had outbreaks during the study. Compared to non-pilot care homes, there was no evidence of significant difference in the proportion of homes with outbreaks, or the size of outbreaks. Qualitative data showed difficulty implementing testing strategies due to excessive work burden. Factors influencing adherence related to test integration and procedural factors, socio-economic factors, cognitive overload and the emotional value of testing.

**Conclusion:**

Implementation of staff and visitor care home LFD testing protocols was poorly adhered to and consequently did not reduce the number or scale of COVID-19 outbreaks. More focus is needed on the contextual and behavioural factors that influence protocol adherence.

## Key Points

- It is difficult to implement rigorous biweekly staff testing within already over-burdened care homes.
- Adherence to the testing protocol was poor, due to many multifactorial reasons, including contextual and human factors.
- Implementation of these testing strategies did not significantly reduce the number or size of COVID-19 outbreaks in care homes.
- To achieve protocol adherence, staff would have to sacrifice essential care duties, which could facilitate COVID-19 spread.

## Introduction

Care homes have been severely affected by COVID-19 [1–3] because residents are at high risk of both infection with SARS-CoV-2 and more severe COVID-19 disease [2, 4]. The addition of testing for infected individuals to infection prevention control (IPC) measures may reduce the risk of transmission and help manage outbreaks. The diagnostic standard for identification of clinical cases of COVID-19 are laboratory-based polymerase chain reaction (PCR) tests [5, 6]. In England, at the time of this study, the standard protocol had been to PCR test residents monthly, or between times if they became symptomatic; and to PCR test staff weekly, or if symptomatic. Visitors were not tested, and only outdoors visits were permitted, unless it was an end-of-life situation, when indoor visits with appropriate personal protective equipment (PPE) were allowed.

PCR testing has been affected by delays in testing and reporting, creating windows in which infected individuals were not identified and could spread the virus, or leading to unnecessary isolation of residents and staff [7]. Deployment of rapid on-site tests could improve the speed and scale of testing. Such tests need not only to be sufficiently accurate but also rapid, safe to use outside a laboratory [8], easy to administer and fit with the IPC workflow of the care home [7]. Antigen-based lateral flow devices (LFDs) have been deployed nationally in care homes, but not yet evaluated in an end-to-end workflow context.

We deployed LFDs as part of a community testing pilot within the Liverpool City Council (LCC) area of England, giving us an opportunity to understand these issues in greater depth [9, 10]. We aimed to evaluate outcomes in terms of preventing outbreaks, and process through the adoption of and adherence to the LFD testing regimens. We sought to understand behavioural, usability, administrative and organisational factors that might affect the testing process and its impact on COVID-19 prevention.

## Methods

Staff were tested using LFDs twice weekly, based on the epidemiology that the highest risk of transmission is within five days of symptom onset [11]. LFD tests were self-administered. A PCR test was performed simultaneously alongside the second test each week as part of the existing PCR testing regime in care homes. We used the Innova SARS-CoV-2 antigen rapid LFD, which is currently in widespread use across UK care homes. The performance of this test has been assessed in the Liverpool population when attending asymptomatic testing centres [12]. Visitor testing protocols stipulated that two negative LFD tests within a 24-hour period were provided prior to care home visits. The first test took place at a city centre site, where a trained nurse observed self-swabbing technique. Concurrent PCR testing was performed as quality assurance. If the LFD was negative, then a second LFD test (within 24 hours and undertaken by care home staff) was taken before visiting was allowed. If either LFD test result was positive, the visitor was asked to immediately self-isolate according to Government guidelines and request a confirmatory PCR test.

We undertook a descriptive epidemiological analysis of COVID-19 testing and case data, alongside a qualitative exploratory study with care home staff members involved in testing. All 86 care homes within the LCC region were approached to take part in the study. Upon enrolment, care home staff were trained by members of the armed forces, who had delivered LFD testing during the Liverpool community testing pilot. The study was undertaken between the 1 December 2020 and the 10 January 2021.

All staff and visitor test results from study care homes were compiled alongside routine resident testing data (once monthly or symptomatic PCR testing). The prevalence of COVID-19 was recorded up to 10 days after the study period ended, and epidemic curves were created for homes with positive cases. An outbreak was defined as two or more confirmed or clinically suspected cases of COVID-19 with onset dates within 14 days of each other [13]. Prevalence of outbreaks within the study homes was compared to non-study care homes in the LCC region using Fisher’s exact test, and the size of outbreak compared using the Mann–Whitney U test. Adjustment for sociodemographic factors was not applied due to sample size limitations.

Adherence to the testing protocol was evaluated by: (i) identifying number of staff members for each home that took part in the scheme compared to the reported number of staff employed at each home; (ii) calculating the testing ratio between LFD and PCR tests (which would be 2:1 if the protocol was adhered to) and (iii) reporting the total number of LFD tests performed by each staff member. If there was 100% adherence, then each staff member would have performed 12 tests (2 weekly tests for 6 weeks).

Qualitative methods were applied to understand the user experience with LFD testing and contextual factors affecting testing [14]. Participants were initially approached by email and this was followed-up, as appropriate, with a presentation letter, a participant information sheet and a consent form. Semi-structured interviews were conducted remotely, using videoconferencing platforms (Zoom and Microsoft Teams). Participants who accepted to take part in the study were asked to return the signed consent forms. Interviews lasted 30 to 45 minutes on average. Interviews were audio and video recorded on an encrypted device, with the consent of participants. The interview protocol consisted of a selection of questions covering: participant demographics and experience with testing for COVID-19; pathway and process of use, including training experience; errors and unexpected situations; the ideal user profile and use cases; overall comments on ‘trust in use’; and recommendations for future uses.

Interviews were transcribed verbatim using a software for automatic generation of written transcription (Otter.ai) and coded using NVivo (v.12, QSR). Data were thematically analysed and findings reported using consolidated criteria for reporting qualitative research (COREQ) [15]. Framework Analysis Method (FAM) was used to organise the data and to support thematic analysis [16]. Findings, consisting of main themes and quotes, were presented to participants in an ‘on-line’ event, during which participants were encouraged to ask questions about the themes identified, the process of investigation and the research outcome.

## Results

### Epidemiological analysis

**Figure 1:**
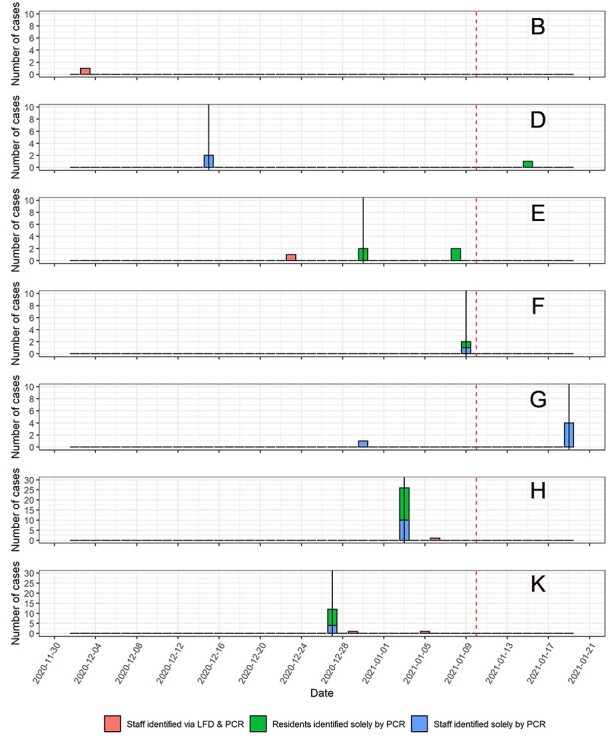
Epi-curves of care homes in the COVID-19 testing pilot scheme (Black vertical lines represent the date that an outbreak was declare. Red dashed vertical lines represent the end of the testing scheme).

Eleven care homes agreed to participate. All were outbreak-free at the start of the study. By the end of the study period, seven out of the eleven homes had identified COVID-19 positive individuals amongst one or more residents or staff. Six homes had entered outbreak (two or more staff or residents infected) within 10 days after the study period (Figure 1). Cases were identified through the pilot scheme, through pre-existing standard resident testing, and through testing of staff not following the test protocol (i.e. they had a PCR test but did not perform any LFD tests). In only one out of the six outbreak homes was a positive LFD result identified before the outbreak. The remaining homes’ index cases were identified solely through PCR.

Out of the 75 care homes in Liverpool who were not part of the study, four were in outbreak at the start of the study and were excluded from further analysis. There was no statistical difference in the proportion of outbreaks observed during the study period (odds ratio 2.1; 95% CI 0.5–9.4%; *P* = 0.32) between pilot care homes (54.5%; 95% CI 23.4–83.3%, 6/11) and other care homes (36.6%; 95% CI 25.5–48.9%, 26/71). There was no statistical difference in the size of outbreak amongst residents and staff (*P* = 0.42) between pilot homes (median 0%, range 0–38.8%, *n* = 6) and other homes (median 0%, range: 0–64.8%, *n* = 26). There was no statistical difference (Mann–Whitney *U* Test, *P* = 0.58) between the size (total residents and staff) of pilot (median 82, range: 44–136) and other (median 79, range: 9–364) care homes.

**Figure 2:**
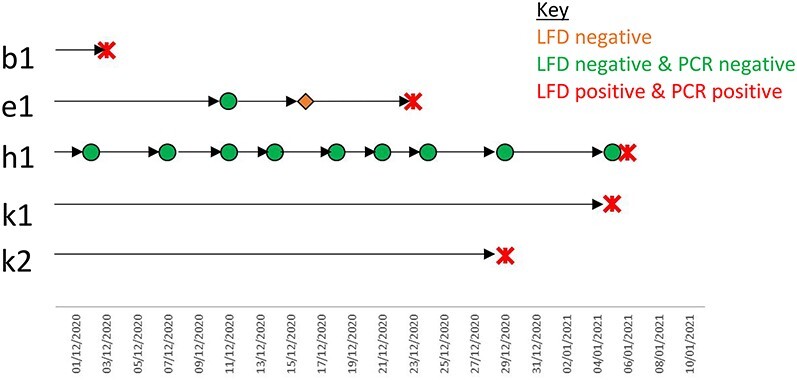
Adherence of testing protocol of the five COVID-19 positive individuals in a care home testing pilot scheme.

During the study, 1,638 LFD tests were performed, of which 828 had matched PCR tests. The resultant prevalence was 0.31 (95% CI 0.10–0.71) positive tests per 100 LFD tests performed (*n* = 5), and 1.23 (95% CI 0.40–2.84) positive staff members per 100 staff tested via LFD. All positive LFDs results were confirmed by PCR tests. No false-positive or false-negative LFD test results were identified. There were no void or unreadable LFD results recorded. Totally, 11 PCR results were void. Of the five LFD and PCR positive cases, three performed only one LFD test during the study period (Figure 2). The two of other cases (e1 and h1) adhered with the number of LFD tests expected.

**Figure 3:**
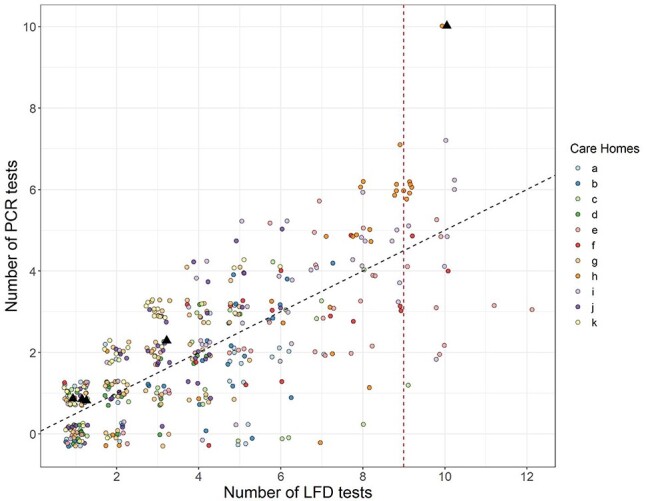
Number of tests performed by care home staff members. Black dashed line equates to a 2:1 testing ratio. Red dashed line represents 75% of expected number of LFDs performed. Black triangles represent positive COVID-19 cases detected through PCR and LFD testing.

The majority of staff participated in the study (81.7%, 407/498), though this was highly variable between homes (Appendix A). The overall testing ratio was 1.98 LFD tests to 1 PCR test, yet only 64 individuals (15.7%) achieved the expected testing ratio of 2:1 (Figure 3).

About, 8.6% of staff members performed more than nine tests ($\ge$75% protocol adherence), 25.3% performed six or more ($\ge$50% adherence), and the majority (62.9%) performed four tests or less (≤25% adherence) (Appendix A). The proportion of staff achieving test adherence varied considerably between homes. There was no apparent trend between testing protocol adherence and outbreak status, and the sample size was too small to effectively stratify the results to perform robust modelling of the data.

Eight out of eleven study care homes participated in visitor testing. One hundred and thirteen care home visitors attended the central testing site. LFD testing identified nine COVID-19 positive individuals, who were then requested to self-isolate according to Government guidelines. Subsequently, PCR testing identified two of these individuals as false positives. One hundred and four individuals tested negative and could proceed to visit a pilot care home. One individual was identified as LFD negative and PCR positive and was informed before arriving at a care home and did not enter it. Of the eligible visitors, 101 arrived at their respective care homes and all tested negative on arrival.

### Human factors analysis

#### Participants

Interviews were conducted with 15 staff members of 9 care homes in the study. A total of 450 minutes of interviews were conducted. Demographics details of participants and care homes characteristics can be found in Table 1.

**Table 1:** Participant and site characteristics

Site characteristics (*n* = 9)	
Type of LTCFs	Number
Nursing homes	4
Residential homes	5
**Number of staff**	**average (min, max)**
Nursing homes	33 (20, 50)
Residential homes	38 (26, 48)
**Number of residents**	**average (min, max)**
Nursing homes	32 (18,46)
Residential homes	45 (27,56)
**Participant characteristics (*n* = 15)**	**Number**
**Gender**	
Male	1
Female	14
**Job role**	
Managerial	11
Senior carer	1
Staff nurse	2
Administrator	1

### Findings

Two main themes were identified, ‘Service integration’ and ‘Social Factors’. **The ‘Service Integration’ theme** describes pragmatic aspects of test integration and procedural factors. The perceived experience with the test and attitudes of staff members are also categorised under this theme, which consists of four subthemes:

#### Administrative tasks associated to testing procedure

LFD test results were required to be registered using dedicated website portals (Government testing portals), for auditing purposes. Each test had to be recorded individually. The log-in procedure had many steps and was time consuming. As the portals were not directly linked with the care home records system, extra staff required to be allocated to administer this. This was exacerbated by variation in the numbers of staff being tested at the same time, due to shift times and availability, thus, slowing down the diagnostic process. Visitor testing required additional staff work. Managing visitors required supervision, to enable a smooth process and to safeguard residents. Visitors not familiar with the procedure and/or with digital technologies struggled to navigate the interface and staff members were frequently asked to support them with this, placing additional pressures and distracting them from other tasks. Most participating homes limited the number of visitors allowed at any one time to enable staff to continue with routine tasks without testing ‘taking over’.

#### Training

Training was provided by army personnel and consisted of a 2-hour live demonstration conducted at the Exhibition Centre in Liverpool. Staff attendees then trained their colleagues (cascade training). The main criticalities associated with the training process were:

1) inconsistencies with training from the army and the test-as-conducted, especially the expected waiting time to read results and correct process of use;
2) unsupervised cascade training; this could create discrepancies in the process
3) trained staff members did not have the chance to directly trial the device during training and no instruction material was left for reference.

As a result, not all staff members were confident in conducting the test after completion of training.

#### Testing pathway and procedures

A critical issue affecting testing was the lack of a standardized process, in part due to inadequate testing areas (e.g. limited space). Care homes had a diverse range of facilities and rooms allocated for testing and equipment. This variability hindered the standardization and potentially affected adherence to the recommended protocol. Staff members were asked to regularly return to the care home for testing. This was often outside their rostered shifts and they were reluctant to comply.

#### Changes in the workload for staff members

Conducting the test for staff members and visitors in accordance with IPC measures and social distancing required significant planning. The combination of LFDs and PCR tests on a regular basis increased pressure on staff, adding to an already saturated workflow, exacerbated by COVID restrictions.

The **‘Social Factors’** theme showed that rapid testing had the potential to enable connections, to reopen care homes to visitors, and to gradually lift restrictions*.* In addition to the impact on family visits, restrictions have also limited visits from GPs and healthcare professionals. The restoration of these visits, with increased healthcare support for residents, and healthcare advice for care home staff, was seen as a potential positive outcome. Despite the lack of clarity about testing procedures and their reliability, staff members indicated that they had joined the pilot with a sense of positivity because of the potential to improve care for residents and family members. Main themes, with descriptions and illustrative quotes, can be seen in Appendix B.

## Discussion

Adherence to LFD testing protocols among staff was poor, with the majority of staff completing less than a third of the tests specified. We found several reasons for this: potential loss of knowledge through cascade training, test regimens complicating workflows of already over-burdened staff, and limited space in care homes to conduct the regimen. Visitor testing drew substantially on staff time and care homes needed to limit visitation to continue to provide routine care duties.

Adherence was poor despite the pilot being rolled out in care homes with an eagerness to participate. A disconnect exists between the prescribed testing regime and the ‘real-life’ context of use. Requirements for staff to get tested multiple times a week were not compatible with the realities of the working schedule of care home employees or employers. Testing regimes designed to increase the probability of detection face significant barriers that will likely amplify existing frustrations with current employment practices. Failure to address this disconnect between testing regimes and the care home workforce risks an increase in staff dissatisfaction and its attendant potential for increased staff turnover and burnout.

The themes identified in the qualitative research were reflected in the adherence data. Many staff members in the pilot care homes did not participate at all, and the majority of those who participated had less than 25% adherence to the study protocol. Additionally, no voided/unreadable LFD tests were recorded against an expected value of between 8 and 275 (i.e. 0.7–16.8% (mean 5.4%) [10]. The lack of void LFD tests is surprising, and the reasons are unknown, but we hypothesise that void results were not uploaded onto the testing system or incorrectly uploaded as negative results, and/or that the swabbing and testing procedures were not followed correctly. This raises further questions about the validity of negative results as the samples collected or data reported may not have been adequate.

The study testing protocol did not prevent outbreaks. Protocol adherence was too poor and sample size was too small to draw any firm conclusions on what protection could be afforded through better implementation of LFD-based screening. All outbreaks identified through the study had a staff member as the index case. In four cases, the index case was identified by PCR. It is not clear whether LFD might have identified the index cases sooner, and thus prevented an outbreak if adherence was higher. In essence, the testing programme did not have the opportunity to work, because it was not implemented as designed.

If visitor testing was to be based on one negative LFD test result, then there is the potential for a false-negative individual entering the care home. In this study, one out of 104 negative LFD results was linked to a positive PCR (1/104, 1.0%). However, quantitative PCR was not used so it is not known whether this individual was likely infectious. Other studies have shown that LFD may miss infectious individuals compared with PCR on the first day of the transmission window; therefore, we adopted a dual LFD in 24 hours approach in this high consequence setting [17]. The risk of a potential false-negative results is numerically small. Nevertheless, decisions on whether this risk is acceptable should recognise that one undetected case could have severe consequences.

The strengths of this study are the bringing together objective and subjective data to study a testing regime, which largely mirrors protocols currently adopted nationally in the UK. There are 14,000 care homes in the UK [18], and the findings may be of limited generalisability but remain important. Our sample over-represents nursing homes and under-represents residential homes, when nursing homes make up approximately a third of all UK care homes [19]. We would, however, expect testing concordance and accuracy of technique to be greater in nursing homes because of the presence of registered staff and the well-recognised role that healthcare experience plays on the accuracy of LFDs [10]. Our sample size was likely too small, and implementation too incomplete, to be able to detect any reduction in transmissions through LFD testing. Such effects will not be observable until the important issues about adherence, and the contextual, organisational, professional and workflow issues that are vital to testing implementation, are addressed.

## Conclusion

The implementation of new testing regimes that include LFDs could provide the possibility of opening up care homes to visitors and to enable the rapid testing of staff to support infection prevention and control efforts. However, we have shown that it is difficult to implement a rigorous testing strategy in already over-burdened care homes. We identified significant issues with test protocol adherence, underpinned by contextual factors, which could undermine the contribution of LFD testing to protecting care home residents. Testing adherence was poor, and the (PCR and LFD) testing protocol as implemented did not prevent care homes from experiencing COVID-19 outbreaks; as such, we question the added value that they provide to care homes. It is important to acknowledge that despite testing protocols enabling staff to ‘restore a sense of normality for residents’ [20], outbreaks still occurred. Testing in itself cannot be seen as a panacea and until the risk of COVID-19 care home outbreaks is significantly reduced, through successful widespread community vaccination, the maintenance of high levels of infection prevention and control (such as the continued use of PPE and limited physical contact) remains paramount [21]. This remains true for both care home staff and visitors. The opportunity costs of testing regimes that add to care home workload need to be taken into consideration in future policy making.

This study highlights the importance of implementation over test performance for staff testing, and the need for more than one negative LFD for visitor testing. Due to the highly vulnerable nature of care home residents, consideration needs to be given to whether a single LFD strategy should be used to facilitate close physical interactions with the highly vulnerable care home resident population. Without addressing the contextual and human factors that lead to poor adherence of testing protocols, these testing regimes will not have the opportunity to perform at the required level to prevent outbreaks in care homes.

## Supplementary Material

aa-21-0610-File005_afab162
