# Supplementary material for: Enhanced lateral flow testing strategies in care homes are associated with poor adherence and were insufficient to prevent COVID-19 outbreaks: results from a mixed methods implementation study
Source: Age Ageing. 2021 Jul 16;50(6):1868–75. doi: 10.1093/ageing/afab162 (PMC8406873; doi:10.1093/ageing/afab162)
Supplement: aa-21-0610-File005_afab162 [file aa-21-0610-file005_afab162.docx]

Enhanced lateral flow testing strategies in care homes are associated with poor adherence and were insufficient to prevent COVID-19 outbreaks: results from a mixed methods implementation study.

**SUPPLEMENTARY DATA**

**Appendix A**

Appendix Table A – Testing protocol and adherence data in Liverpool care homes trialling lateral flow device based testing protocols. (Grey rows are care homes that experienced outbreaks. All visitor and resident LFD results were negative.)

|  | **Information from Liverpool City Council** | | **Testing Protocol Summary** | | | | | | **Testing Adherence and Outbreak Information** | | |
| --- | --- | --- | --- | --- | --- | --- | --- | --- | --- | --- | --- |
| Care Home | Number of Residents | Number of Staff | Number of Staff Tested | Number of Staff LFD Tests Performed | Number of Staff PCR Tests Performed | Number of Staff LFD Positive Cases | Number of Visitor LFD Performed | Number of Resident LFD Performed | >=50% protocol adherence  (1 LFD a week or more) | >75% protocol adherence  (1.5 LFD a week or more) | Size of Outbreak |
| A | 36 | 27 | 35 | 105 | 39 | 0 | 0 | 0 | 11.4% (4) | 0% | N/A |
| B | 35 | 41 | 28 | 113 | 44 | 1 | 22 | 38 | 21.4 % (6) | 0% | N/A |
| C | 33 | 32 | 25 | 109 | 34 | 0 | 1 | 0 | 36.0% (9) | 4.0% (1) | N/A |
| D | 54 | 46 | 30 | 64 | 28 | 0 | 35 | 6 | 0% | 0% | 3 |
| E | 48 | 44 | 41 | 242 | 99 | 1 | 13 | 3 | 56.0% (23) | 19.5% (8) | 4 |
| F | 26 | 18 | 18 | 107 | 48 | 0 | 3 | 6 | 11.1% (10) | 22.2% (4) | 2 |
| G | 30 | 65 | 74 | 207 | 121 | 0 | 0 | 6 | 0% | 0% | 10 |
| H | 41 | 26 | 30 | 209 | 124 | 1 | 7 | 0 | 80.0% (24) | 36.7% (11) | 26 |
| I | 18 | 67 | 45 | 266 | 144 | 0 | 0 | 0 | 53.3% (24) | 24.4% (11) | N/A |
| J | 53 | 83 | 42 | 125 | 75 | 0 | 6 | 1 | 4.8% (2) | 0% | N/A |
| K | 46 | 49 | 39 | 91 | 72 | 2 | 14 | 0 | 2.6% (1) | 0% | 14 |
| **Total** | **420** | **498** | **407** | **1638** | **828** | **5** | **101** | **60** | **25.3% (103)** | **8.6% (35)** |  |

**Appendix B**

Appendix Table B - Main themes and illustrative quotes, regarding utilisation of lateral flow devise based testing protocols in Liverpool care homes.

| **Theme** | **Sub-theme** | **Description** | **Illustrative quotes** |
| --- | --- | --- | --- |
| Service integration | Administrative tasks | Including problems encountered when managing testing procedure (e.g.: registering data to the portal) for staff members and visitors. | “So, if you have five staff turning up in their own time, you don't want them waiting too long. There's a bit of pressure there as well. Do you know what I mean to get in those early. That's why we have extra staff. And we do have an extra member of staff when it comes to tests (10-11)”  “some of the residents are, can be a little bit unsettled during the visit and we don't want them to go and close contact with the loved one. So sometimes we need to be there with them, you know, to reassure [the resident], and explain why this visit is why they can't come into the close contact. It is very, very time consuming, (12).  “So if they put your name in next page, put your database then next page, put your address in next page. So it's probably I think, all in all, it's probably maybe 15 pages of information that they've got to [fill]. We've got elderly relatives, […] they don't do FaceTime, they don't do the internet. So that then added time on for us as we would have to do that registration process, then do the test and wait for 30 minutes for the test […] it's time consuming (13)”.  *“You just have to find the time […] I had to write to my manager to explain them that I wasn't doing everything I needed to do. Because I was doing this pilot, you know (14)”* |
|  | Training | describing the training experiences of staff members, the positive and negative aspects of training. It also describes the ‘cascade training’. | *“I think the information I received when I first went to get my own test on you know, the standard test was good. It's just it's that lack of experience, isn't it of doing that to yourself and understanding you when you start to gag you want to stop and suddenly someone else who kind of carries on into properly. So I think the information given was good. It's just the actual carrying out is not. It's not easy for someone to [swab] themself (3)”.*  *“maybe would be useful if they make a video clips like they did for the PCR test at the beginning we all we didn't know what is about obviously, this this pandemic is unexpected for all of us. So we didn't know how to do these tests and the YouTube videos and then there are other videos that were available (12)”.*  *“The positive results can develop in between 20 minutes to half an hour. And that's what we're doing. That was good that someone highlighted that because it was a bit confusing one. And that wasn't highlighted during training. All that. You see the results five minutes after because it develops really quickly. You still need to observe it and then take out that it hasn't changed from that to positive between that half an hour and good enough to go over from what we've done, the results just stayed the same (1)”.*  *“I'm here now maybe on the weekends, I'm not here and visitors might want to come on the weekend. So every member of staff then you've got your day and your night staff, which is that's important because people were coming they might want to come past six, seven o'clock. So to make sure that All the members of staff have training.”* |
|  | Testing pathway | Describing the current set-up for both staff and visitor testing at care home and implications for optimising future triaging set-ups. | *“Well, our setting is quite modern. And we have quite a lot of rooms and facilities where it's safe for people to do the tests we have, you know, we have clinic areas, and treatment areas available. And we're quite lucky to have quite a few safe areas and the infection control is, is good. So, but I know other care homes probably would struggle, because the older buildings or it's not as clinical, were quite clinical prepared (12)”.*  *“So not many staff been able to do it. Because travel and the and the cost and then their time and obviously this time of the year is everyone busy. You know, they don't want to spend all these hours to come for the lateral flow test (12)”.* |
|  | Workload | Including the anticipated workload and resources required to implement testing into the routine workflow of the staff. | *“So, where you would normally say a social worker would normally come in and do review. And you might be able that social worker would sit, maybe sit in the office and go through the care documentation before taking any of the stuffs time, for instance. Whereas now, we've now got the additional time of scanning an email and all of the documentation of social worker, and then happen to do the video calls to do the assessments that we would maybe wouldn't need to sit in. So, like some assessments can take two, two and a half, maybe three hours. W that normally monopolise that much of our time if it was face to face? (13)”*  *“My workload dramatically suffered, I had to write to my manager, explain what I'm not doing? Yeah, of course, it was just too much (15)”.* |
| Social factors |  | Factors influencing the social interactions of both staff members and residents.  This theme describes the importance of testing to enable visits and to reassure staff members; it also includes emotional factors that affect the natural course of the events. | “We needed the GP to come out and say: 'don't worry about giving them medication (to an old lady), don't worry about giving her anything to eat and drink', and taking that responsibility and stress from the staff. And the GP was expecting us to make that decision, she literally should have just stopped the medication.” *(10)*  *“And seeing my residents so happy. Seeing my residents So happy, […] in the same room without no restrictions, apart from the PPE of seeing and their loved ones, that's, that's been the best for me. You know, there's been plenty of tears the past week, not just residence, it's been staff, it's been all happy tears (6).”* |
